# Sodium ion channel mutations in glioblastoma patients correlate with shorter survival

**DOI:** 10.1186/1476-4598-10-17

**Published:** 2011-02-11

**Authors:** Avadhut D Joshi, D Williams Parsons, Victor E Velculescu, Gregory J Riggins

**Affiliations:** 1 Department of Neurosurgery, Johns Hopkins University Medical School, Baltimore, MD, 21231, USA; 2 Ludwig Center for Cancer Genetics and Therapeutics, and The Howard Hughes Medical Institute at Johns Hopkins Kimmel Cancer Center, Baltimore, MD 21231, USA; 3 Department of Pediatrics, Section of Hematology-Oncology, Baylor College of Medicine, Houston TX 77030, USA

## Abstract

**Background:**

Glioblastoma Multiforme (GBM) is the most common and invasive astrocytic tumor associated with dismal prognosis. Treatment for GBM patients has advanced, but the median survival remains a meager 15 months. In a recent study, 20,000 genes from 21 GBM patients were sequenced that identified frequent mutations in ion channel genes. The goal of this study was to determine whether ion channel mutations have a role in disease progression and whether molecular targeting of ion channels is a promising therapeutic strategy for GBM patients. Therefore, we compared GBM patient survival on the basis of presence or absence of mutations in calcium, potassium and sodium ion transport genes. Cardiac glycosides, known sodium channel inhibitors, were then tested for their ability to inhibit GBM cell proliferation.

**Results:**

Nearly 90% of patients showed at least one mutation in ion transport genes. GBM patients with mutations in sodium channels showed a significantly shorter survival compared to patients with no sodium channel mutations, whereas a similar comparison based on mutational status of calcium or potassium ion channel mutations showed no survival differences. Experimentally, targeting GBM cells with cardiac glycosides such as digoxin and ouabain demonstrated preferential cytotoxicity against U-87 and D54 GBM cells compared to non-tumor astrocytes (NTAs).

**Conclusions:**

These pilot studies of GBM patients with sodium channel mutations indicate an association with a more aggressive disease and significantly shorter survival. Moreover, inhibition of GBM cells by ion channel inhibitors such as cardiac glycosides suggest a therapeutic strategy with relatively safe drugs for targeting GBM ion channel mutations. **Key Words: **glioblastoma multiforme, ion channels, mutations, small molecule inhibitors, cardiac glycosides.

## Background

Glioblastoma multiforme (GBM) is a malignant astrocytic brain tumor with a current median survival of about 15 months [1]. The current standard of care therapy is surgery followed by concurrent radiation plus temozolomide. The addition of the DNA alkylating agent temozolomide improves the survival by 10 weeks. There is a similar increase in survival with local delivery of BCNU, the other currently approved chemotherapy for this tumor [2]. The survival gains for GBM patients show that progress can be made, but this progress has been slow. It is not clear if large survival gains can be achieved with the current trio of radiation, surgery and DNA damaging chemotherapy.

Recently, GBMs have undergone a large-scale mutation screen [3] and the molecular targets for this cancer can be re-evaluated. Critical to this approach is the identification of altered proteins or pathways that initiate and/or promote tumor growth. Ideally, these molecular targets are unique to the tumor cell, and therapy specific to the alteration does not harm normal cells. There are some very well known genes mutated in GBM such as the tumor suppressors *p53 *and *PTEN*, and amplification or mutation of the *EGFR *and *PDGFRA *oncogenes. Unfortunately, molecular targeting efforts in GBM so far have not been translated into clinical success, despite some promising results of targeted therapy in a few other cancers.

Although there are many possible reasons why molecular targeting has not yet been successful in GBM, it is possible that different or additional molecular targets in combination will have better success. A recent survey of the coding sequence of 20,661 genes in GBM genomes has implicated many new mutated genes [3]. Similar to other cancers there are many mutated genes in GBM and these genes cluster into key pathways or gene groups. This clustering occurs more than chance predicts, suggesting that these are a small number of key cellular processes that need to be altered in the majority GBMs. One cluster of mutated genes reported by Parsons et al. [3] was the ion channel genes. Of the 555 genes involved in sodium, potassium, calcium and other ion transport, 55 mutations were detected affecting 90% of the samples studied with at least one somatic mutation. The statistical significance of this observation was estimated to be p < 0.001 and the ion channels were ranked as one of the top gene clusters implicated by acquired mutations in GBM.

Ion channels form a crucial part of cellular machinery and are responsible for transporting essential ions across cell membranes, maintaining cell shape, cell volume and plasma membrane potential [4-6]. Recent evidence suggests a role for ion channels in cancer progression and metastasis [7-9]. Ion channels, such as sodium channels, potassium channels and calcium channels, have been implicated for their role in a number of different cancers such as colon cancer, prostate cancer, breast cancer and lung cancer [8,9]. For example, the up-regulation of voltage gated sodium channels is associated with progression of breast cancer metastasis [10].

In this study, we report a correlation between ion channel mutations and patient survival. Twenty-one GBM patients where sodium, potassium and calcium channel gene sequences were known [3] were analyzed further for this study. GBM patients with a mutation in any of the sodium channel genes had a significantly shorter survival compared to those with wild-type sequence. In contrast, there was no statistical survival difference for GBM patients with either potassium or calcium channel mutations. We extended these findings with a preliminary *in vitro *laboratory investigation to determine if known sodium channel inhibitors had an effect on GBM cells relative to cells with a normal genome.

## Patients and Methods

### Patient Characteristics

The twenty-one patients included in this study were those analyzed for mutations in 20,661 genes in a previous study [3]. There were eight females and 13 males. Median age of GBM patients was 45 years (median age in males 45 years, median age in females 60 years; not significantly different). None of the GBM patients had received a prior chemo or a radiation therapy. One of the 21 patients was diagnosed as a ganglioglioma patient but was associated with a later recurrence of GBM. Median survival of the patients was 54.9 weeks (range 2 - 215 weeks). The characteristics of patient cohort are summarized in Table 1.

**Table 1:** GBM patient characteristics

Tumor ID	Patient age (years)	Sex	Pathology	Recurrent GBM	Secondary GBM	Prior radiation therapy	Prior chemotherapy	Survival after tumor sample obtained (Days)	Sample type	Sodium channel mutations	Potassium channel mutations	Calcium channel mutations
Br02X	39	M	GBM	No	No	No	No	Unknown	XG	Yes	No	Yes

Br03X	44	M	GBM	No	No	No	No	422	XG	Yes	Yes	No

Br04X	45	F	GBM	Yes	No	NA	NA	Unknown	XG	No	No	No

Br05X	41	M	GBM	No	No	No	No	563	XG	No	No	Yes

Br06X	11	M	GBM	No	No	No	No	986	XG	No	No	No

Br07X	45	M	GBM	No	No	No	No	350	XG	Yes	No	Yes

Br08X	54	M	HGG	No	No	No	No	384	XG	No	Yes	No

Br09P	51	M	GBM	No	No	No	No	588	PT	Yes	Yes	Yes

Br10P*	30	F	GBM	No	No	No	No	813	PT	No	No	Yes

Br11P*	32	M	GBM	No	No	No	No	1502	PT	No	Yes	No

Br12P*	31	M	GBM	No	No	No	No	566	PT	No	No	Yes

Br13X	59	F	GBM	No	No	No	No	174	XG	Yes	Yes	Yes

Br14X	61	F	GBM	No	No	No	No	Unknown	XG	No	Yes	Yes

Br15X	61	M	GBM	No	No	No	No	56	XG	Yes	No	Yes

Br16X	63	M	GBM	No	No	No	No	Unknown	XG	Yes	No	Yes

Br17X	63	M	GBM	No	No	No	No	964	XG	No	Yes	Yes

Br20P	77	F	GBM	No	No	No	No	122	PT	Yes	Yes	Yes

Br23X	78	F	GBM	No	No	No	No	16	XG	Yes	No	No

Br25X	45	M	GBM	No	No	No	No	48	XG	Yes	No	No

Br26X	66	F	GBM	No	No	No	No	61	XG	No	Yes	No

Br29P	42	F	HGG	Yes	NA	NA	NA	Unknown	PT	Yes	Yes	No

*Patients with IDH1 mutations.

### GBM Patient Samples and Genome Sequencing

The GBM sequencing results have been previously published by Parsons et al. [3]. GBM patient tumor samples were obtained using an IRB approved protocols. Twenty-one GBM samples consisting of six surgically resected patient tumors and 15 samples were passaged in nude mice as xenografts. These 21 samples were amplified by PCR for sequence analysis. Primer pairs were used to amplify and sequence 175,471 coding exons and adjacent intronic splice donor and acceptor sequences in 21 GBM samples and one matched normal sample as described previously [3].

### Gene Selection

All the mutated genes were classified according to gene ontology into different gene sets [3]. Ion channel classification used in this study included voltage gated ion channels and ion co-transporters and are referred to as channels for sake of simplicity. All the genes involved in ion transport including, sodium channels, potassium channels, calcium channels were selected and genes specifically associated with sodium channels, potassium channels and calcium channels were selected individually from these sets.

### Cell Culture

U-87 GBM cells were obtained from ATCC and D54 GBM cells were obtained from Duke University Medical Center. Both U87 and D54 cells were maintained in DMEM supplemented with 10% FBS and penicillin/streptomycin. Non-tumor astrocytes (NTAs) consisted of brain cortex tissue surgically resected from epilepsy patients using an IRB approved protocol. The cortex was cut into fine pieces using a scalpel and was cultured for less than 8 passages in DMEM F12 cell culture medium supplemented with 10% FBS and penicillin/streptomycin.

### Cell Proliferation and Drug Sensitivity Assay

The cardiac glycosides digoxin and ouabain (Sigma-Aldrich) were dissolved in methanol and phosphate buffered saline (PBS), respectively, to make a stock solution of 25 mM. Subsequent dilutions were made from this stock solution. Methanol and PBS were used as a vehicle control for digoxin and ouabain respectively, during the proliferation assay. Cell proliferation was assessed using an alamarBlue^® ^assay (Invitrogen, Carlsbad, CA). U-87, D54 and NTAs were plated (1000 cells/well) in black clear bottom 96 well plates (Becton Dickinson, Bedford MA) and incubated overnight. The following day, each drug was added at its designed concentration with 20 μl of 10X alamarBlue reagents. The volume in each well was made up to 200 μl with the growth medium. After 72 hours incubation, alamarBlue fluorescence was measured on a Perkin Elmer Wallac 1420 Multilabel counter (Perkin Elmer, Turku, Finland) with a 540 nm excitation filter and a 590 nm emission filter. Fold inhibition was calculated by dividing the fluorescence values for control cells (cells treated with vehicle) with fluorescence values of cells treated with a particular concentration of cardiac glycosides. For apoptosis analysis, both U-87 and NTAs were plated in a six well plate (100,000 cells/well) and incubated for 24 hours. After 24 hours, cells were treated with 500 nM of digoxin and ouabain overnight and observed under a microscope

### Statistical Analysis

GraphPad Prism 5 software was used to compute all the survival curves. To determine the clinical outcome, patient survival was used as a measure where survival was defined as the time in days from first surgical resection of GBM to death. Out of 21 patient samples, survival data were available for 16 different patients.

## Results

### Mutations in Sodium Ion Channels are Associated with Shorter Survival in GBM Patients

Systematic analyses of functional gene groups and pathways from a previous study [3] identified ion channel genes that transport sodium, potassium or calcium ions as one of the gene groups most frequently mutated in GBM. The sodium, potassium and calcium ion transport gene groups were each evaluated to determine if mutations in these gene groups altered average patient survival. Nineteen of the 21 patients (90%) showed at least one mutation in sodium, potassium or calcium channels taken together. Fourteen sodium channel genes (Table 2), 13 potassium channel genes (Table 3) and 18 calcium channel genes (Table 4) had somatic mutations. None of the mutations were found in more than one patient except for SCN9A, CACNA1H, and TRPV5 where each gene was mutated in two patients. Interestingly, all the samples with IDH1 mutations did not have any sodium channel mutations. A comprehensive list of genes was divided into individual lists of genes that were associated with sodium channels, potassium channels or calcium channels (Tables 2, 3 and 4). For example, patients were classified into the sodium channel mutation group if they had a mutation in at least one sodium channel gene (listed in Table 2). If there were no mutations in any sodium channels, the patients were grouped into a sodium channel 'unmutated' group. Patients were grouped in a similar way for potassium channels and calcium channels.

**Table 2:** Sodium channel mutations

Gene	Transcript Accession	Tumor	Nucleotide (genomic)	Nucleotide (cDNA)	Amino acid (protein)	Mutation Type
ATP12A	NM_001676	Br13X	g.chr13:24178566C>G	c.2134C>G	p.Q712E	Missense

SCN1B	CCDS12441.1	BR02X	g.chr19:40213563C>T	UTR-2C>T	5'UTR	5'UTR

SCN3A	NM_006922	Br03x	g.chr2:165772558G>A	c.5612G>A	p.R1871 Q	Missense

SCN3B	CCDS8442. 1	Br16X	g.chr11:123018465_12301 8464delCT	c.344_345del CT	fs	INDEL

SCN5A	NM_000335	Br20P	g.chr3:38579032G>T	c.3838G>T	p.V1280 F	Missense

SCN9A	NM_002977	Br9PT	g.chr2:166987822G>A	c.583G>A	p.V195I	Missense

SCN9A	NM_002977	Br07X	g.chr2:166881761C>T	c.4862C>T	p.T1621 M	Missense

SLC11A1	CCDS2415. 1	Br25X	g.chr2:219077407G>A	c.520G>A	p.V174I	Missense

SLC1A2	NM_004171	Br03X	g.chr11:35239082_352390 81delCT	c.1660_1661 delCT	fs	INDEL

SLC5A7	CCDS2074. 1	Br15X	g.chr2:108067164C>A	c.263C>A	p.P88Q	Missense

SLC8A1	CCDS1806. 1	Br07X	g.chr2:40568624C>G	c.448C>G	p.L150V	Missense

SLC9A1	CCDS295.1	Br07X	g.chr1:27120218C>T	c.1006C>T	p.L336F	Missense

SLC9A2	CCDS2062. 1	Br23X	g.chr2:102732730G>A	c.479G>A	p.R160H	Missense

SLC9A4	NM_001011 552	Br9PT	g.chr2:102583058G>A	c.1201G>A	p.V401I	Missense

TRPM5	NM_014555	Br29P	g.chr11:2382782G>A	c.3459G>A	p.W1153 X	Missense

**Table 3:** Potassium channel mutations

Gene	Transcript Accession	Tumor	Nucleotide (genomic)*	Nucleotide (cDNA)	Amino acid (protein)	Mutation Type
ATP12A	NM_001676	Br13X	g.chr13:24178566C>G	c.2134C>G	p.Q712E	Missense

GRIK4	CCDS8433.1	Br17X	g.chr11:120343207G>A	c.2360G>A	p.G787E	Missense

KCNA4	NM_002233	Br29P	g.chr11:29990620C>T	c.182C>T	p.S61F	Missense

KCNB2	CCDS6209.1	Br11P	g.chr8:74011087G>A	c.943G>A	p.A315T	Missense

KCND2	CCDS5776.1	Br03X	g.chr7:119979914C>T	c.1597C>T	p.R533X	Missense

KCNG3	CCDS1809.1	Br26X	g.chr2:42631881G>A	c.412G>A	p.D138N	Missense

KCNH1	CCDS1496.1	Br9PT	g.chr1:207365704G>T	c.1662G>T	p.K554N	Missense

KCNH5	CCDS9756.1	Br08X	g.chr14:62316201C>T	c.2017C>T	p.R673W	Missense

KCNJ15	CCDS13656.1	Br03X	g.chr21:38593138G>A	c.85G>A	p.V29I	Missense

KCNK1	CCDS1599.1	Br26X	g.chr1:230109198G>A	c.478G>A	p.V160I	Missense

LRRC4B	ENST00000253728	Br03X	g.chr19:55713328C>T	c.809C>T	p.T270I	Missense

REN	NM_000537	Br20P	g.chr1:200862821G>A	c.226G>A	p.V76M	Missense

SLC12A5	CCDS13391.1	Br14X	g.chr20:44097883G>A	c.340G>A	p.V114I	Missense

**Table 4:** Calcium channel mutations

Gene	Transcript Accession	Tumor	Nucleotide (genomic)*	Nucleotide (cDNA)	Amino acid (protein)	Mutation Type
ATP2B1	CCDS9035.1	Br12P	g.chr12:88531401T>A	c.502T>A	p.L168I	Missense

CACNA1A	NM_000068	Br15X	g.chr19:13184518C>T	c.5980C>T	p.P1994S	Missense

CACNA1C	NM_000719	Br17X	g.chr12:2094747C>A	c.146C>A	p.A49D	Missense

CACNA1H	NM_021098	Br15X	g.chr16:1199144C>T	c.3475C>T	p.Q1159X	Missense

CACNA1H	NM_021098	Br05X	g.chr16:1201735G>A	c.4495G>A	p.V1499M	Missense

CACNA2D3	NM_018398	Br07X	g.chr3:54905889G>A	c.2320G>A	p.A774T	Missense

CHRNA3	CCDS10305.1	Br14X	g.chr15:76681620T>C	c.419T>C	p.L140S	Missense

CHRNA9	CCDS3459.1	Br15X	g.chr4:40197220C>T	c.1195C>T	p.R399C	Missense

GRIN2B	CCDS8662.1	Br13X	g.chr12:13608123G>A	c.3316G>A	p.E1106K	Missense

GRM1	CCDS5209.1	Br15X	g.chr6:146761918C>T	c.2050C>T	p.R684C	Missense

ITPR3	CCDS4783.1	Br16X	g.chr6:33761264G>A	c.5458G>A	p.E1820K	Missense

KIAA0703	NM_014861	Br20P	g.chr16:83006699G>A	IVS5+1G>A	Splice Site	Splice Site

NMUR1	CCDS2486.1	Br20P	g.chr2:232219206C>T	c.31C>T	p.L11F	Missense

PKD1L2	NM_182740	Br14X	g.chr16:79765857G>T	c.2747G>T	p.G916V	Missense

RYR2	NM_001035	Br10P	g.chr1:233820306G>A	c.256G>A	p.V86M	Missense

RYR2	NM_001035	Br17X	g.chr1:233858930G>A	c.365G>A	p.R122H	Missense

RYR3	NM_001036	Br9PT	g.chr15:31893017C>T	c.10447C>T	p.R3483W	Missense

SLC8A1	CCDS1806.1	Br07X	g.chr2:40568624C>G	c.448C>G	p.L150V	Missense

STIM2	CCDS3440.1	Br02X	g.chr4:26686687G>T	c.1544G>T	p.R515L	Missense

TRPV5	CCDS5875.1	Br15X	g.chr7:142139519G>A	c.1064G>A	p.R355H	Missense

TRPV5	CCDS5875.1	Br14X	g.chr7:142139507G>A	c.1076G>A	p.R359H	Missense

**Figure 1:**
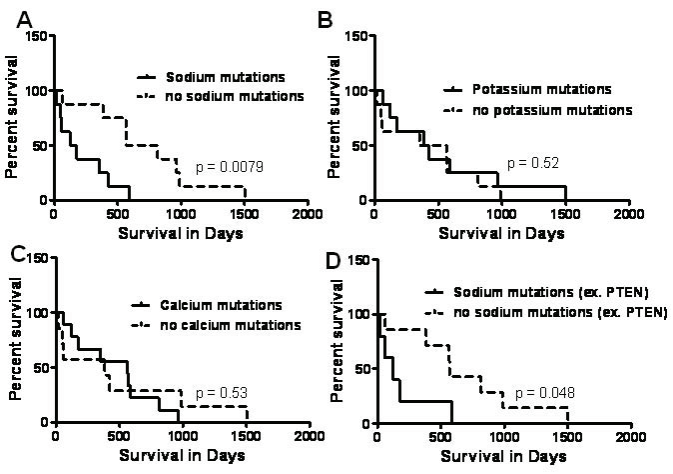
**Sodium ion channel mutations predict shorter survival in GBM**. **A: **Survival of GBM patients was compared in patients with mutated and unmutated sodium channels using log rank test. Analysis revealed that GBM patients with sodium channel mutations (n = 8) had a significantly shorter survival (p = 0.0079) compared to patients with no sodium channel mutations (n = 8). **B: **GBM patient survival was compared on the basis of potassium channel mutations. There was no significant difference in median survival of patients with (n = 8) and without (n = 8) potassium channel mutations. **C: **Analysis of calcium channels also failed to show any significant difference in survival of GBM patients when compared on the basis of presence (n = 9) or absence (n = 7) of calcium channel mutations. **D: **In order to exclude the effect of PTEN mutations on survival of GBM patients with sodium channel mutations, patients with PTEN mutation were excluded from the analysis and the survival curves compared on the basis of presence or absence of sodium channel mutations. Exclusion of PTEN did not have any effect and GBM patients with sodium channel mutations (n = 5) showed a significantly shorter survival (p = 0.048) compared to patients with no sodium channel mutations (n = 7).

To determine the role of individual ion channels in GBM patients, survival of GBM patients was compared using Kaplan Meier analyses. GBM patients with sodium channel mutations showed a significantly shorter survival (p = 0.0079) compared to patients with unmutated sodium channels. Median survival of GBM patients with mutated sodium channels was 148 days compared to 689 days in patients with no sodium channel mutations (Figure 1A). A similar comparison in GBM patients with mutated and unmutated potassium channels (Figure 1B), and mutated and unmutated calcium channels (Figure 1C) showed no significant difference in survival. These observations suggest that survival difference seen in GBM patients with mutated and unmutated sodium channels are not random.

GBM patients with PTEN mutations are associated with shorter survival [11]. Therefore, to rule out the effect of PTEN mutation on the survival curves with mutated and unmutated sodium channels, we excluded the patients with PTEN mutations. In spite of the exclusion of patients with PTEN mutation, GBM patients with sodium channel mutations were associated with significantly shorter survival (Figure 1D). Median survival of patients with sodium channel mutations was 122 days compared to 566 days in patients with no mutations.

### Targeting Ion Channels Preferentially Inhibits Growth of Glioblastoma Cells

Because sodium channel mutations had a substantial effect on GBM patient survival, targeting sodium channels may be an effective way to counter GBM cell growth. We started our study with sodium channel inhibitors with previous clinical use. Based on information in the literature, and a larger screen of libraries of approved drugs (data not shown), we selected two cardiac glycosides, digoxin (FDA approved) and ouabain, to test on GBM cells. Our reasoning for choosing cardiac glycosides was based on two main previously reported findings. First, the anti-proliferative or anticancer effect of cardiac glycosides is well documented [12-14] and second; cardiac glycosides may be neuroprotective [15] and thus, might be used safely in the central nervous system.

**Figure 2:**
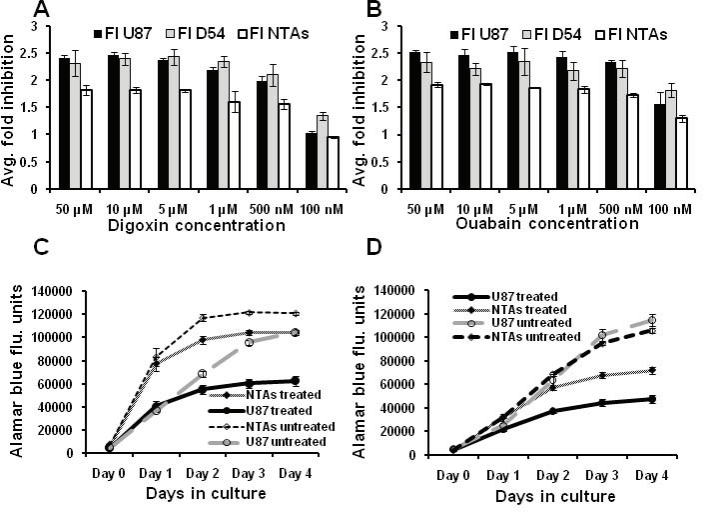
**Cardiac glycosides preferentially target GBM cells over non tumor astrocytes (NTAs)**: U-87, D54 GBM cells and NTA cells when treated with different concentrations of cardiac glycosides demonstrated a significantly higher cytotoxicity (p < 0.05) against GBM cells at concentrations ranging from 50 μM to 500 nM. At concentrations of 100 nM and below there was no significant cytotoxic effect. **A: **Bar graph demonstrating preferential cytotoxicity of Digoxin against U-87 and D54 GBM cells **B: **Bar graph demonstrating preferential cytotoxicity of Ouabain against U-87 and D54 GBM cells. **C and D: **Growth curves of U-87 GBM and NTAs were plotted for four days using an alamarBlue based assay demonstrate preferential inhibition of U-87 GBM cells compared to NTAs when treated with 500 nM of Digoxin (2C) and Ouabain (2D).

**Figure 3:**
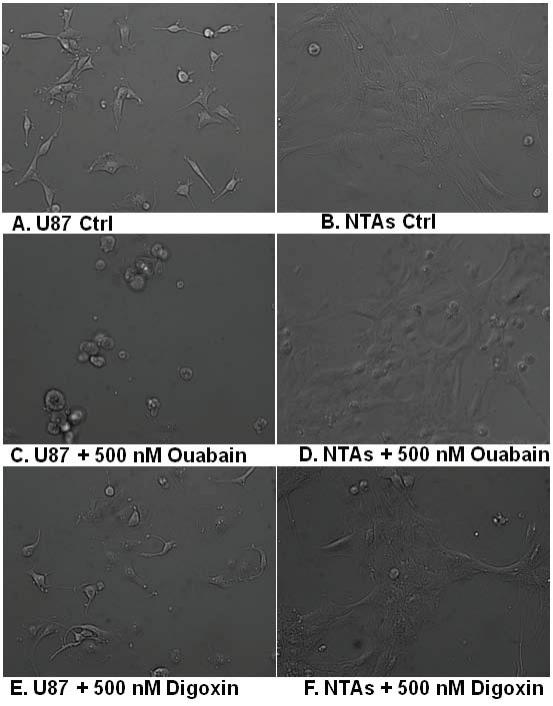
**Light microscope pictures depicting apoptotic phenotype/dead U-87 GBM cells after an overnight treatment with ouabain and digoxin**. Non-tumor astrocytes remain unaffected for most part, although they demonstrated delayed (after 36 hrs) apoptotic phenotype.

The effect of ouabain and digoxin on proliferation of U-87 and D54 GBM cells and NTAs was tested first, using an alamarBlue based assay. Cells were treated at different concentrations ranging from 10 nM to 50 μM in a 96 well plate. After 72 hours, both digoxin and ouabain showed preferential anti-proliferation and toxicity against U-87 and D54 GBM cells compared to the NTAs (Figure 2A and 2B). In addition, comparison of growth curves of U-87 and NTAs treated with 500 nM digoxin and ouabain demonstrated a preferential inhibition of U-87 GBM cells over NTAs (Figure 2C and 2D). Furthermore, to confirm that GBM cells were preferentially targeted by an alternative technique, U-87 cells and NTAs treated with 500 nM of digoxin and ouabain overnight and were observed under a light microscope next morning. Figure 3 demonstrates that U-87 GBM cells treated with digoxin and ouabain detach and showed an apoptotic phenotype, whereas NTAs remained adherent and did not show an apoptotic phenotype, confirming the preferential cytotoxicity of cardiac glycosides.

## Discussion

In the GBM patients studied those with mutations in the sodium ion channel genes had a significantly shorter survival compared to patients without a mutation. In comparison, similar analyses of mutations in potassium channels and calcium channels showed no statistical survival differences. One biological possible explanation for this observation is that sodium channel mutations promote GBM tumor growth and/or invasion, thereby decreasing survival, whereas other non-sodium ion channel mutations do not function to alter invasion. This is the first report suggesting a possible role of ion channel mutations in GBM prognosis. Nineteen out of 21 (90%) patient samples showed at least one mutation in sodium, potassium or calcium channels. It will be important to see if this observation can be reproduced in larger studies and/or other patient populations. Furthermore, it was found that patients with no sodium channel mutations were younger (median age 43 years) compared to patients with mutated sodium channel mutations (median age 51 years), although the difference did not turn out to be statistically significant (p = 0.117).

Recently, IDH1 mutations have been identified to be associated with a specific subgroup of GBM patients who are younger and have a better prognosis [3,16]. Interestingly, we found that all the patients with IDH1 mutations were a part of sodium channel unmutated group. However it is not known whether this association is significant because of the small sample size. It also raises the question whether IDH1 mutations would contribute to improved survival in patients with unmutated sodium channels. Analysis of survival data after excluding IDH1 mutated patients revealed that median survival in patients with sodium channel mutations was 148 days compared to 563 days in sodium channel unmutated patients in accordance with our earlier observations, however the p value dropped to 0.06 (data not shown). These observations warrant a larger and more in depth study to investigate whether there is an association between IDH1 mutation and GBM patients with unmutated sodium channels and whether the improved survival seen in GBM patients with unmutated sodium channels is independent of IDH1 mutation status.

Ion channel genes were mutated at a higher frequency compared to other genes (p < 0.001). Moreover, individual groups of genes consisting of calcium ion transport (p < 0.001), sodium ion transport (p < 0.005) and potassium ion transport (p = 0.037) showed a significantly higher frequency of mutation [3]. Most of the ion channel genes were mutated only once except for SCN9A, CACNA1H and TRPV5 which were each mutated twice in the set of 21 patients. Since no gene was mutated more than twice in this set and there are many possible ion channels with mutations, this indicates a low mutation rate for each individual gene, despite the group being highly mutated. Subset classes of the ion channels, such as SCN or SLC (both sodium channels) or KCN (potassium channels), were also enriched for mutations [3]. These observations suggests that mutations in a gene family or molecular pathway of similar function when considered in combination may be more informative than a single gene, when evaluating tumor growth and selecting molecular targets.

In this report, we have used the mutation status of sodium channels as a variable for comparing patient survival. However, which mutations are relevant to GBM biology and how they alter the clinical course of GBM remains unknown. One interpretation of our data is that sodium channel inhibition slows tumor cell growth, suggesting that sodium channel mutations are activating, or activate some mechanism responsible for poor prognosis. However, there is no evidence at the molecular level as to how these mutations might work. Further in depth molecular physiological studies to determine the direct effect of the mutations on membrane potential and polarization/depolarization and cell signaling of the tumor cells would be an option to study this question.

Sodium, potassium and calcium channels form an intricate network that maintains ionic balance in the cell and mutation in any one of the ion channels could alter many cellular functions. One hypothesis is that ion channel mutations are partially responsible for the increased motility of GBM cells. Voltage gated sodium channels have already been implicated for their role in enhancing the invasiveness of breast cancer and prostate cancer [10,17-19]. Higher expression of SCN5A has been associated with higher metastatic potential. It has also been reported that EGF may increase metastatic potential of prostate cancer by up regulation of SCN9A [19]. Although, our data does not describe expression levels of sodium channels, there is a possibility that the mutations in SCN5A and SCN9A may cause an increase in the activity of sodium channels thereby increasing the metastatic potential of GBM and decreasing survival of the patients.

Ion channels might be investigated as a pharmacological target for GBM patient therapy. Our data demonstrates that ion channel inhibitors, cardiac glycosides in this case can preferentially inhibits GBM cells over non-tumor astrocytes (NTAs) when tested *in vitro*. There is no evidence yet that cardiac glycosides molecularly interact directly with any of the mutated sodium channel (shown in Table 2). Nevertheless, preferential targeting of GBM cells by cardiac glycosides suggests that ion channels can be targeted and should be evaluated as a therapeutic drug target for treating GBM in the future. Attempts have been made to treat GBMs with cardiac glycosides with modified structures that reduce their cardio-toxicity and increase anti-proliferative capability [20]. Similarly, targeting of α1 subunit of the sodium pump using the siRNA inhibited growth and migration of lung cancer cells [21]. Voltage gated sodium channels have also been targeted in prostate cancer cells with encouraging results [22].

## Conclusion

In summary, we have shown that mutations in sodium channels are associated with an aggressive form of GBM. We also show *in vitro *growth inhibition by ion channel inhibitors, suggesting that GBM might be targeted using ion channel inhibitors. These observations from different lines of investigation hint that sodium ion channels should be investigated further as a molecular therapeutic target in GBM.

## Competing interests

The authors declare that they have no competing interests.

## Authors' contributions

AJ analyzed the mutation data on the basis of ion channel mutations, made the Kaplan Meier survival curves, performed the cytotoxicity assays using digoxin and ouabain and wrote the manuscript. DWP generated all the mutation data for the GBM genome project. VV conceived the genome sequencing study and critiqued the manuscript. GJR conceived and help design the study, wrote the manuscript and critically revised the manuscript. All authors have read and agreed to submit the manuscript.
